# Investigating the shared genetic structure between rheumatoid arthritis and stroke

**DOI:** 10.1186/s41065-025-00386-8

**Published:** 2025-02-14

**Authors:** Qian Qin, Yong’An Jiang, Hengyi Fan, Raorao Yuan, Bo Zhong, Yichen Zhang, Zile Zhang, Xin Lei, Jianhui Cai, Shiqi Cheng

**Affiliations:** 1 Department of Neurosurgery, the Second Affiliated Hospital, Jiangxi Medical College, Nanchang University, Nanchang, 330006 Jiangxi P. R. China; 2 Nanchang University, Nanchang, 330006 Jiangxi P. R. China; 3 Department of Critical Care Medicine, Second Affiliated Hospital, Jiangxi Medical College, Nanchang University, Nanchang, 330006 Jiangxi China; 4 Department of Neurosurgery, Xinyu People’s Hospital, Xinyu, 338000 Jiangxi P. R. China; 5 Department of Neurosurgery, Nanchang County People’s Hospital, Nanchang, 330200 Jiangxi P. R. China; 6 Nanchang Cranio-Cerebral Trauma Laboratory, Nanchang, 330200 Jiangxi P. R. China

**Keywords:** Rheumatoid arthritis, Stroke, Genome-wide association studies, Shared genetic structure, Multi-trait association analysis

## Abstract

**Background:**

Rheumatoid arthritis (RA) increases the risk of stroke. However, the relationship between RA and stroke remains unclear. This study aimed to explore the shared genetics architecture (i.e., common genetic basis between different traits, diseases, or phenotypes) of RA and stroke, aiming to improve the intervention and management of patients with RA and stroke.

**Methods:**

Pooled statistics from publicly available genome-wide association studies for RA (8,255 cases and 409,001 controls) and stroke (43,132 cases and 43,132 controls) were used. A genome-wide positive association was conducted to (examine the comprehensive effects of genetic variants on a particular trait, disease, or phenotype at the genome-wide scale). Local genetic correlation studies used linkage disequilibrium score regression and super genetic covariance analyzer. Single nucleotide polymorphisms (SNPs) at risk were identified using genome-wide association study multiple trait analysis and PLINK software (*P*_*snp*_ <5e-08), followed by functional localization and annotation using Functional Mapping and Annotation of Genome-Wide Association Studies to identify specific genes and genetic variants that may contribute to the disease. Finally, a transcriptome-wide association study explored the relationship between genes and their association with RA risk.

**Results:**

A genome-wide significant positive correlation was evident between RA and stroke (genetic correlation = 0.3756). Among the localized genomic regions, the correlation between RA and stroke in the region of chr2:201572564–202,829,668 was the most significant (*p* = 0.0015). We identified 179 significant SNPs and five common risk genes for RA and stroke (IRF5, RNASET2, ZNF438, UBE2LS, and SYNGR1). These genes are involved in the immune-inflammatory pathway.

**Conclusions:**

The findings suggest a shared genetic structure between RA and stroke. These findings may provide new insights into RA and stroke pathogenesis, and contribute to the development of new diagnostic markers and therapeutic targeted drugs to improve the clinical outcomes of patients with RA and stroke.

**Supplementary Information:**

The online version contains supplementary material available at 10.1186/s41065-025-00386-8.

## Background

Stroke, including ischemic stroke and hemorrhagic stroke, is characterized by the sudden loss of focal neurological function and is a major public health issue [1]. In 2019, stroke was the second-leading cause of mortality worldwide. Stroke can lead to significant disability, reduce the life expectancy and quality of life of patients, and impose a heavy physical and emotional toll on patients and their families [2, 3]. Stroke is associated with hypertension, obesity, hyperlipidemia, smoking, and substance abuse. All these can significantly shorten the life span of patients [4–7]. Other risk factors associated with stroke have also been actively explored.

Rheumatoid arthritis (RA) is a chronic inflammatory autoimmune disease affecting 0.5–1% of the global population [8]. RA causes inflammation and structural damage to joints, resulting in reduced mobility, increased disability, and reduced quality of life [9]. Although the exact etiology of RA remains unclear, significant advances in its treatment have been reported in recent years. Despite these advances, patients still experience poor outcomes in terms of quality of life, cure rates, and mortality compared to the general population [10].

Research on RA and stroke is increasing. RA is a significant contributor to an elevated risk of stroke due to specific risk factors and underlying mechanisms [11]. A growing body of research supports the comorbidity of RA and stroke, suggesting a potentially shared etiology influenced by genetic factors. Simultaneously, exploring the shared genetic structure—a common genetic basis between different traits, diseases, or phenotypes—could help improve risk prediction, early intervention, and management of patients with RA and stroke.

Recent publications have addressed the genetics of RA and stroke. Okada et al. [12] performed a genome-wide association study (GWAS) on RA and identified 42 novel risk single nucleotide polymorphisms (SNPs), including rs909685 (located in SYNGR1), rs110896379 (located in UBE2L3-YDJC), and rs793108 (located in ZNF438). The findings provided new directions for exploring the pathogenesis of RA [12]. A multi-progenitor meta-analysis identified 34 novel loci (*P* < 5e-8) and newly discovered candidate genes, including tumor necrosis factor alpha-induced protein 3 (TNFAIP3) interacting protein 2 (TNIP2) and TNF receptor superfamily member 11 A (TNFRSF11A), confirming the important roles of the immune system and joint tissues in the etiology of RA [13].

**Fig. 1:**
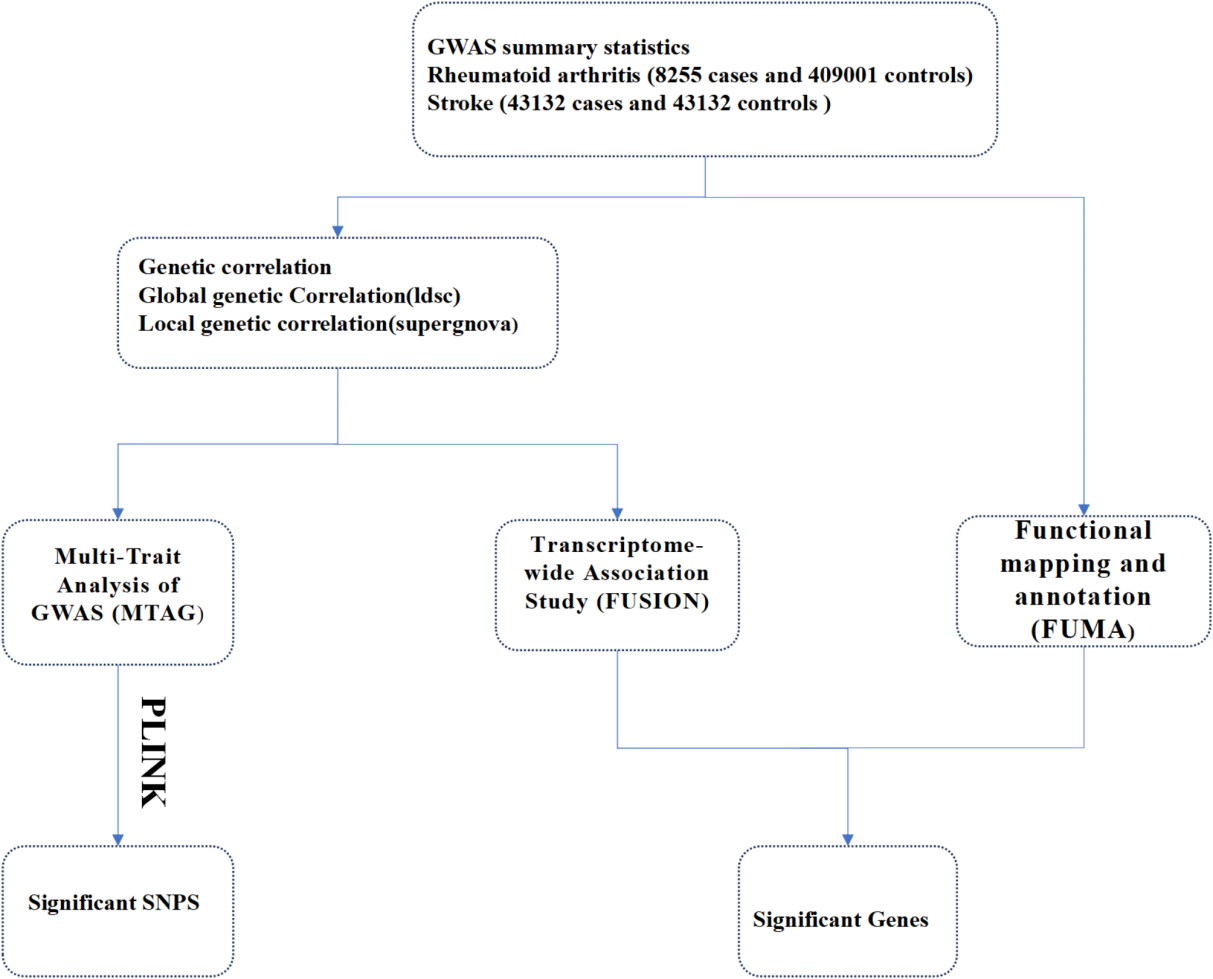
Analytical strategy of the study

In this study, we explored the shared genetic structure between RA and stroke using extensive GWAS data, estimating their genetic correlations, and identifying possible shared SNPs and candidate genes through cross-trait meta-analysis and functional analyses (Fig. 1). The findings offer novel insights into the underlying mechanisms and inspire the development of new diagnostic markers and therapeutic targets.

## Methods

### Data sources

We obtained GWAS summary statistics for RA (8,255 cases, 409,001 controls) from the European Genetic Research Program (HG19/GRCH37) and for stroke (43,132 cases, 43,132 controls) from the Finnish Genetic Research Program (R10). All samples were from European populations. Supplemental Table S1 lists the specific characteristics of the datasets used in this study. When processing GWAS data, we filtered out SNPs with minor allele frequencies < 1% and removed duplicate or missing SNPs.

### Genome-wide genetic correlation analysis

Linkage disequilibrium score regression (LDSC) is a commonly used genetic correlation analysis method for estimating genetic contributions to complex diseases and traits. LDSC is mainly used to infer the strength of association between each SNP and complex traits by estimating the LD score of each SNP [14]. In this study, we used LDSC in Python 2.7 to derive heritability estimates for RA and stroke. Additionally, we calculated the genetic correlation (r_g_) between the two diseases by quantifying the shared genetic variance relative to the square root of SNP heritability estimates. We also used the 1000 Genomes Europe reference dataset to convert the GWAS summary statistics into pre-calculated LD scores to finalize the genetic correlation between RA and stroke.

### Localized genetic correlation analysis

Local genetic correlation quantifies the genetic similarity of complex traits in a specific genomic region and is another important approach for addressing the potential etiological mechanisms shared by multiple complex traits [15, 16]. However, accurately estimating local genetic correlations remains challenging because of LD in local genomic regions and sample overlaps across studies. Super genetic covariance analyzer (SUPERGNOVA) is a combinatorial framework for various types of genetic correlation analyses [17]. SUPERGNOVA provides statistically rigorous and computationally efficient inference of genome-wide and local genetic correlations, with significant advantages when applied to local genomic regions [18]. In this study, we used the SUPERGNOVA method to investigate local genetic correlations between RA and stroke.

### Cross-feature meta-analysis

Multi-trait analysis of GWAS (MTAG) is a method for jointly analyzing the summary statistics of GWAS for different traits (possibly overlapping samples) to determine trait-specific genetic associations. Compared to many existing multi-trait methods, MTAG provides the significant advantage of being applicable to the summary statistics of GWAS for an arbitrary number of traits, generating estimates of trait-specific effects for each SNP. Therefore, we used MTAG in Python 2.7 to identify risk SNPs associated with RA and stroke by setting the genome-wide significance level of MTAG to P_mtag_ < 5e-8. To further validate our hypothesis of a shared genetic structure between RA and stroke, we calculated the upper bound of the false discovery rate (FDR). We then used PLINK software (https://www.cog-genomics.org/plink/1.9) to cluster them [19]. Independent SNPs significantly associated with the phenotype were identified by applying specific parameters through clustering: -clump-kb 500 / -clump-p1 5e-12 / - clump-p2 1e-08 / -clump-r^2^ 0.2.

### SNP annotation

We used the Functional Mapping and Annotation (FUMA; https://fuma.ctglab.nl/) online platform to functionally map and annotate MTAG summary data for major SNPs, associated risk loci, and candidate genes for RA and stroke.

 [20]. FUMA helps identify candidate genes by linking genetic variants to their potential biological functions, tissues of expression, and related pathways, providing insights into how these variants may contribute to the development of RA and stroke.

### Transcriptome-wide association studies (TWAS)

TWAS use transcriptional regulation (expression) as a mediator between genetic variation (genotype) and phenotype, converting the association of a single genetic variant with a phenotype into the association of a gene transcript with a phenotype. These studies are mainly used to determine tissue-specific gene-trait associations [21]. In this study, FUSION software was used to analyze tissue-associated TWAS data from GWAS summary data [22]. The analysis of TWAS used pre-calculated gene expression weights in conjunction with GWAS summary statistics to identify the associations between genes and diseases. Whole blood data from the Genotype-Tissue Expression (GTEx) consortium were used as a reference panel in combination with GWAS summary statistics for TWAS [22]. These risk genes were then compared to those obtained from the FUMA to identify potential risk genes.

## Results

### Genome-wide genetic correlations

We included 417,256 SNPs associated with RA and 340,999 SNPs associated with stroke in the LDSC analysis. Single-trait LDSC showed SNP heritability estimates of 0.0114 (standard error [SE] = 0.0016) for GWAS_RA_ and 0.0215 (SE = 0.0021) for GWAS_stroke_. The paired LDSC results indicated a significant genome-wide positive association between RA and stroke (r_g_ = 0.3756, SE = 0.0798, *p* = 2.4998e-6) (Supplemental Table S2).

### Localized genetic correlations

**Fig. 2:**
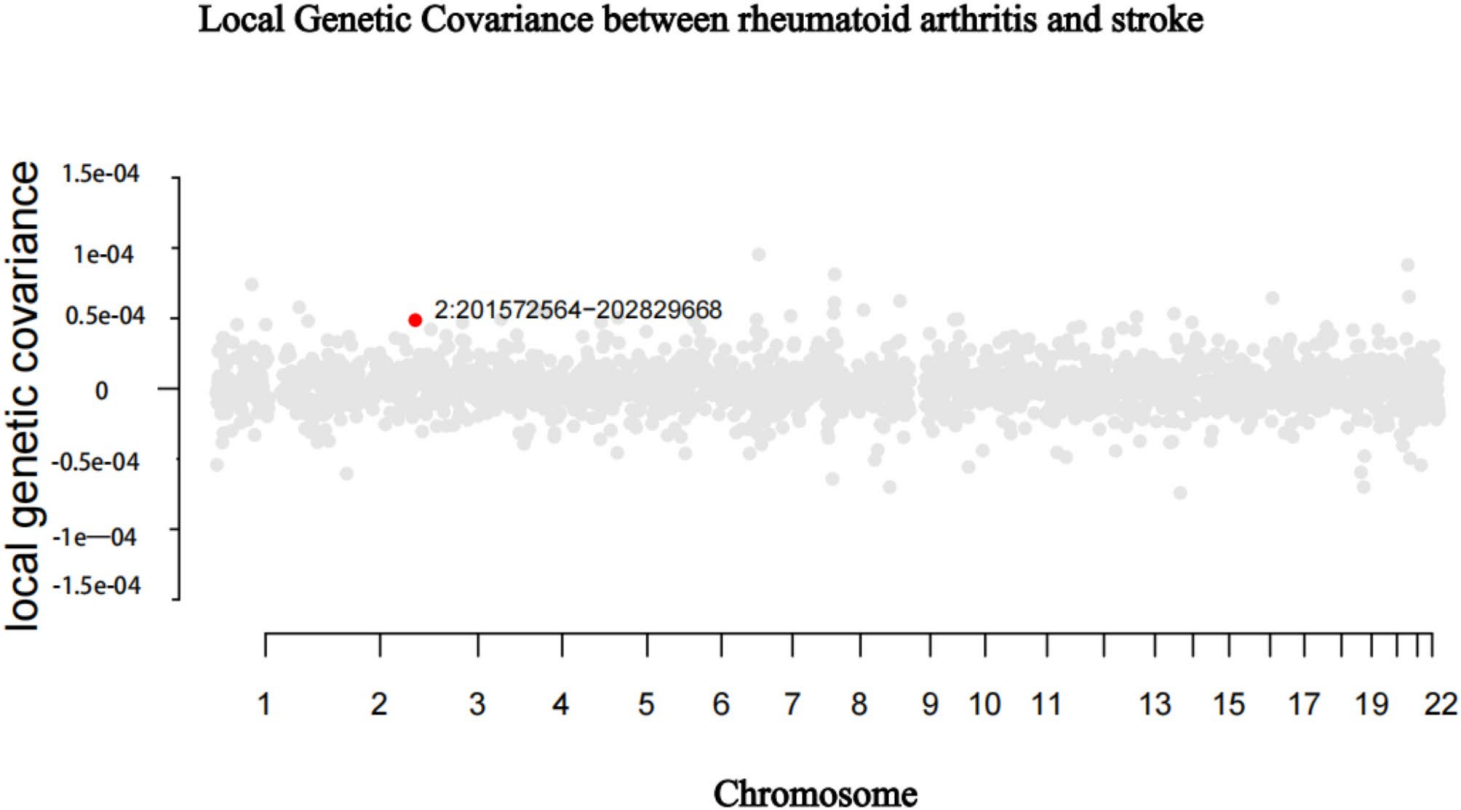
Local genetic correlation between RA and stroke

Considering the significant genome-wide genetic correlations between RA and stroke, we examined specific genomic regions with localized genetic correlations. Using SUPERGNOVA, we identified a significant local genetic correlation between RA and stroke. The strongest local correlation between RA and stroke was chr2:201572564–202829668) (*p* = 0.0012) (Fig. 2, Supplemental Table S3).

### Cross-feature meta-analysis

GWAS_RA_ and GWAS_stroke_ number dramas were entered into the MTAG for cross-trait association analysis. The results of the MTAG analysis provided evidence for the existence of shared genetic loci for RA and stroke. Moreover, the maximum FDRs of 0.03 and 0.04 based on the MTAG of RA and stroke indicated no overall inflation due to violation of the homogeneity assumption (Supplemental Table S4), ruling out the possibility of false positives. Further searching for significant SNPs using P_mtag_<5e-8 yielded 9,683 SNPs with genome-wide significance by GWAS_RA_ and 596 SNPs with genome-wide significance by GWAS_stroke_. Subsequent screening using PLINK identified fi179 of the most significant SNPs (Supplemental Table S5). Concurrent analysis of RA and stroke MTAG summary data for functional localization and annotation identified 61 lead SNPs and 30 risk loci (Supplemental Table S6-7).

### Transcriptome wide association studies (TWAS)

TWAS was performed using whole blood data collected by the GTEx consortium to explore the relationship between genetically predicted mRNA levels and disease risk [23]. This analysis identified 17 genes expressed in the blood that were associated with RA (Supplemental Table S8) and one gene, proline/serine-rich coiled-coil protein 1 (PSRC1), which is also expressed in blood and associated with stroke (Supplemental Table S9). Five overlapping genes obtained from the TWAS and FUMA analyses were identified as potential risk genes (Table 1): interferon regulatory factor (IRF5), ribonuclease T2 (RNASET2), zinc finger protein 438 (ZNF438), ubiquitin-binding enzyme E2L3 (UBE2L3), and synaptic protein 1 (SYNGR1).

**Table 1:** Shared pleiotropic genes linking rheumatoid arthritis and stroke

Gene ID	Gene name	CHR	Gene start	Gene end	IndSigSNPs	minGwasP	TWAS.*P*
ENSG00000128604	IRF5	7	128,577,666	128,590,089	rs3757387	1.466e-13	5.11e-08
ENSG00000026297	RNASET2	6	167,342,992	167,370,679	rs3777722;rs2769343;rs3798307;rs2236313;rs1079145;rs9459839;rs2247314;rs162294	8.974e-12	1.52e-08
**ENSG00000183621**	**ZNF438**	**10**	**31,109,136**	**31,320,866**	**rs1776616**	**2.423e-09**	**8.53e-08**
**ENSG00000185651**	**UBE2L3**	**22**	**21,903,736**	**21,978,323**	**rs5754100**	**9.204e-09**	**8.15e-06**
**ENSG00000100321**	**SYNGR1**	**22**	**39,745,930**	**39,781,593**	**rs5757628**	**7.58e-10**	**1.84e-06**

## Discussion

To the best of our knowledge, this study is the first genome-wide cross-trait analysis to systematically assess the shared genetic structure of RA and stroke using GWAS data. Genome-wide and localized genetics confirmed a significant positive genetic correlation between RA and stroke, consistent with our hypothesis that there are shared genetic pathways and potential loci. We identified 179 SNPs associated with RA and stroke, including SNP rs2476601.

Rs2476601 has been associated with various disorders, including RA [16, 24, 25] and systemic lupus erythematosus [26–28]. Recent studies have shown that SNP rs2476601 is localized to the PTPN22 gene, which encodes a specific lymphatic phosphatase belonging to the tyrosine phosphatase family that inhibits T cell signaling, which in turn alters the inflammatory state of the soft tissues of the joints, leading to RA [23, 29]. However, the effect of SNP rs2476601 on stroke remains unclear. We found that rs2476601 can modulate the alteration of cytokine profiles toward a pro-inflammatory state [30], which in turn alters the inflammatory state of the cerebral vasculature and increases the risk of stroke.

We identified five potential risk genes: IRF5, RNASET2, ZNF438, and UBE2L3, SYNGR1. IRF5 is widely expressed in dendritic cells, monocytes, and B cells. This transcription factor regulates the transactivation of genes related to inflammation and immune response [31]. Furthermore, IRF5 can alter macrophage status by upregulating pro-inflammatory genes and inhibiting anti-inflammatory mediators, inducing immune-related disorders such as RA [32]. Moreover, IRF5 expression can regulate the pro-inflammatory response of microglia and post-stroke inflammation, potentially affecting the recovery of stroke patients. Therefore, we hypothesized that IRF5 mutations are associated with stroke development.

In addition to IRF5, other genes have been studied in recent years. RNASET2, the only human member of the Rh/T2/S acid hydrolase family, contributes to cytoskeletal reorganization and caspase activation in response to oxidative stress [33, 34]. The expression of RNASET2 has been associated with cancer, autoimmune diseases, biological stress, apoptosis, and triggering of innate immunity [35–38]. GWAS data have confirmed an association between RNASET2 and susceptibility to vitiligo, RA, Graves’ disease, and Crohn’s disease [39]. A previous genome-wide meta-analysis involving Korean and European populations identified two new RA susceptibility genes (UBASH3A and SYNGR1). ZNF438 is a transcriptional repressor commonly expressed in 18 different types of tissue, including the brain and heart [40]. We hypothesize that its overexpression may increase the risk of stroke. UBE2L3 is an E2 ubiquitin-coupled enzyme required for activating nuclear factor-kappa B via a linear ubiquitin chain assembly complex downstream of CD40L and TNF-α [41, 42]. The UBE2L3 haplotype is strongly associated with systemic lupus erythematosus and several autoimmune diseases [41, 43]. The relationship between these risk genes, RA, and stroke requires further analysis using larger sample sizes and functional experiments.

Our study has some limitations. First, the data were derived from individuals of European ancestry, limiting the study scope and generalization to other ethnic groups. Second, due to limited data availability, we were unable to perform phenotype-specific analyses of stroke, such as hemorrhagic versus ischemic stroke, nor analyze separate datasets for individuals with these two conditions. Finally, although we identified genes associated with RA and stroke, future longitudinal and experimental studies are necessary to understand the underlying biological mechanisms.

## Conclusion

A comprehensive evaluation of the shared genetic structure between RA and stroke identified 179 significant SNPs and five shared risk genes concerning the genetic relationship between RA and stroke. These findings may aid in the development of novel diagnostic biomarkers, providing new directions for early screening and personalized treatment. Through further functional studies, the roles of these genes can be translated into precise treatment strategies to help improve clinical outcomes in patients with RA and stroke.

## Electronic supplementary material

Below is the link to the electronic supplementary material.

Supplementary Material 1

## Data Availability

All raw data from this study are freely available, and any questions can be directed to the corresponding authors.
