# Supplementary material for: Investigating the shared genetic structure between rheumatoid arthritis and stroke
Source: Hereditas. 2025 Feb 14;162:23. doi: 10.1186/s41065-025-00386-8 (PMC11827134; doi:10.1186/s41065-025-00386-8)
Supplement: Supplementary file 1 — Supplementary Material 1 [file 41065_2025_386_MOESM1_ESM.docx]

# Supplemental Table S1. Details of GWAS Summary Data Sources.

| **Phenotype** | **N-case** | **N-contral** | **Ethnicity** | **N_SNPs** |
| --- | --- | --- | --- | --- |
| Rheumatoid arthritis | 8255 | 409001 | European | 24175266 |
| Stroke | 43132 | 43132 | European | 19216180 |

SNP:single nucleotide polymorphisms

**Table S2.Single-trait LDSC of RA GWAS and stroke GWAS.**

| **Trait 1-Trait 2** | **RA-STROKE** |
| --- | --- |
| r_g_ | 0.3756 |
| standard error(r_g_) | 0.0798 |
| *P* value | 2.4998e-06 |
| Intercept (genetic correlation) | 0.3756 (0.0798) |
| Lambda GC (Trait 1) | 1.0812 |
| Intercept (Trait 1) | 1.0176 (0.0083) |
| Lambda GC (Trait 2) | 1.188 |
| Intercept (Trait 2) | 1.0698 (0.0086) |
| Total Observed scale h2  (Trait 1) | 0.0114 (0.0016) |
| Total Observed scale h2  (Trait 1) | 0.0215 (0.0021) |

**Supplementary Table S3. Summary of local genetic correlations between RA and STROKE.**

| **Chr** | **Start** | **End** | **Number of SNPs** | **local_rhog** | ***P*** |
| --- | --- | --- | --- | --- | --- |
| 2 | 201572564 | 202829668 | 375 | 5.7819785838043634e-05 | 0.001192368162652852 |

SNP: single nucleotide polymorphisms. The start and end described the base pair.

**Supplementary Table S4. Summary of MTAG results.**

| **Trait** | **SNPs** | **N** | **GWAS meanχ**2 | **MTAG mean χ**2 | **Max FDR** |
| --- | --- | --- | --- | --- | --- |
| RA | 8375872 | 417256 | 1.257 | 1.260 | 0.03 |
| STROKE | 19216180 | 340999 | 1.118 | 1.125 | 0.04 |

**Supplementary Table S5. 179SNPS of GWASRA-STROKE results by PLINK( clustering: -clump-kb 500 / -clump-p1 5e-12 / - clump-p2 1e-08 / -clump-r2 0.2.)**

| **CHR** | **F** | **SNP** | **BP** | **P** | **TOTAL** |
| --- | --- | --- | --- | --- | --- |
| **6** | **1** | **rs35139284** | **32561370** | **2.39e-277** | **874** |
| **6** | **1** | **rs9268833** | **32428062** | **3.96e-152** | **834** |
| **6** | **1** | **rs9271365** | **32586794** | **3.79e-121** | **327** |
| **6** | **1** | **rs1281944** | **32597379** | **7.17e-120** | **39** |
| **6** | **1** | **rs2395191** | **32447085** | **2.11e-106** | **167** |
| **6** | **1** | **rs34539464** | **32560339** | **3.33e-105** | **134** |
| **6** | **1** | **rs33964890** | **32583919** | **6.07e-103** | **45** |
| **6** | **1** | **rs112574398** | **32658610** | **4.83e-98** | **273** |
| **6** | **1** | **rs1130456** | **32627379** | **5.81e-96** | **75** |
| **6** | **1** | **rs28383322** | **32592796** | **4.46e-91** | **104** |
| **6** | **1** | **rs58667488** | **32431785** | **1.23e-90** | **1** |
| **6** | **1** | **rs28746860** | **32635033** | **1.53e-86** | **12** |
| **6** | **1** | **rs6927022** | **32612397** | **1.73e-84** | **43** |
| **6** | **1** | **rs9271539** | **32590028** | **2.58e-71** | **122** |
| **6** | **1** | **rs28357082** | **32558176** | **3.2e-71** | **0** |
| **6** | **1** | **rs9272226** | **32602396** | **1.45e-65** | **17** |
| **6** | **1** | **rs146482045** | **31367052** | **1.01e-63** | **307** |
| **6** | **1** | **rs560607175** | **32593316** | **4.61e-59** | **76** |
| **6** | **1** | **rs9380294** | **32380909** | **2.91e-56** | **48** |
| **6** | **1** | **rs3135369** | **32387221** | **5.43e-55** | **180** |
| **6** | **1** | **rs146003472** | **32390751** | **3.08e-54** | **2** |
| **6** | **1** | **rs9469233** | **32688753** | **3.64e-52** | **357** |
| **6** | **1** | **rs9267857** | **32196569** | **2.5e-50** | **32** |
| **6** | **1** | **rs3132972** | **32230354** | **1.34e-49** | **187** |
| **6** | **1** | **rs9271369** | **32586992** | **2.1e-46** | **36** |
| **6** | **1** | **rs9265878** | **31313033** | **5.04e-46** | **238** |
| **6** | **1** | **rs9266271** | **31326082** | **1.65e-45** | **111** |
| **6** | **1** | **rs9266633** | **31346929** | **6.03e-45** | **87** |
| **6** | **1** | **rs1694114** | **32651978** | **4.3e-43** | **30** |
| **6** | **1** | **rs6462** | **32006597** | **1.77e-42** | **18** |
| **6** | **1** | **rs6910879** | **32560739** | **2.57e-41** | **4** |
| **6** | **1** | **rs2273017** | **32337630** | **2.67e-39** | **33** |
| **6** | **1** | **rs1265888** | **32066447** | **3.15e-39** | **16** |
| **6** | **1** | **rs3817967** | **32361469** | **4.15e-38** | **256** |
| **6** | **1** | **rs62404120** | **32612962** | **1.16e-37** | **99** |
| **6** | **1** | **rs2894381** | **32682228** | **1.58e-37** | **26** |
| **1** | **1** | **rs2476601** | **114377568** | **2.68e-34** | **33** |
| **6** | **1** | **rs1004095** | **32153409** | **3.66e-33** | **1** |
| **6** | **1** | **rs567977131** | **31329511** | **5.45e-33** | **3** |
| **6** | **1** | **rs17208188** | **32195005** | **1.57e-32** | **16** |
| **6** | **1** | **rs3763326** | **32413557** | **2.91e-32** | **24** |
| **6** | **1** | **rs137893789** | **32055486** | **3.42e-32** | **16** |
| **6** | **1** | **rs147425711** | **32329883** | **5.12e-32** | **25** |
| **6** | **1** | **rs9275602** | **32682812** | **7.04e-32** | **8** |
| **6** | **1** | **rs2273019** | **32306419** | **1.96e-31** | **31** |
| **6** | **1** | **rs9271719** | **32593479** | **4.69e-31** | **4** |
| **6** | **1** | **rs3749946** | **31448862** | **9.25e-30** | **4** |
| **6** | **1** | **rs139395255** | **31449736** | **1.77e-29** | **8** |
| **6** | **1** | **rs2516466** | **31414327** | **1.9e-29** | **29** |
| **6** | **1** | **rs28688207** | **32628660** | **2.23e-29** | **46** |
| **6** | **1** | **rs2596557** | **31380852** | **2.84e-29** | **154** |
| **6** | **1** | **rs388629** | **32105512** | **3.1e-29** | **104** |
| **6** | **1** | **rs3873375** | **31251360** | **4.9e-29** | **143** |
| **6** | **1** | **rs4248814** | **31346755** | **1.06e-28** | **51** |
| **6** | **1** | **rs17500510** | **32712818** | **2.44e-28** | **0** |
| **6** | **1** | **rs805262** | **31628733** | **5.08e-28** | **51** |
| **6** | **1** | **rs3129294** | **33084671** | **7.07e-28** | **122** |
| **6** | **1** | **rs77639798** | **31509043** | **7.76e-28** | **38** |
| **6** | **1** | **rs3128947** | **32965062** | **1.35e-27** | **1** |
| **6** | **1** | **rs909267** | **31746548** | **7.85e-27** | **32** |
| **6** | **1** | **rs7739491** | **30979203** | **1.32e-26** | **9** |
| **6** | **1** | **rs513095** | **32287876** | **3.05e-26** | **27** |
| **6** | **1** | **rs3130922** | **31461089** | **4.47e-26** | **35** |
| **6** | **1** | **rs9267548** | **31675985** | **6.74e-26** | **29** |
| **6** | **1** | **rs115550566** | **31183907** | **4.99e-25** | **15** |
| **6** | **1** | **rs12663103** | **32161324** | **6.28e-24** | **28** |
| **6** | **1** | **rs9274498** | **32633941** | **1.99e-23** | **12** |
| **6** | **1** | **rs115042889** | **31236035** | **2.11e-23** | **3** |
| **6** | **1** | **rs28869508** | **32343735** | **2.62e-23** | **7** |
| **6** | **1** | **rs4713471** | **31472823** | **2.73e-23** | **7** |
| **6** | **1** | **rs3104395** | **32685426** | **3.63e-23** | **152** |
| **6** | **1** | **rs419132** | **32210799** | **1.35e-22** | **13** |
| **6** | **1** | **rs61309148** | **30792248** | **1.68e-22** | **10** |
| **6** | **1** | **rs2621344** | **32773278** | **2.22e-22** | **14** |
| **6** | **1** | **rs2567279** | **33046662** | **2.71e-22** | **86** |
| **6** | **1** | **rs9265882** | **31313101** | **3.14e-22** | **37** |
| **6** | **1** | **rs3873444** | **32682724** | **3.69e-22** | **0** |
| **6** | **1** | **rs9262559** | **31007304** | **1.84e-21** | **9** |
| **6** | **1** | **rs7742274** | **32746306** | **2.11e-21** | **64** |
| **6** | **1** | **rs17576984** | **32212985** | **2.29e-21** | **0** |
| **6** | **1** | **rs2395108** | **32203608** | **4.77e-21** | **9** |
| **6** | **1** | **rs3128980** | **31402837** | **7.86e-21** | **108** |
| **6** | **1** | **rs3025650** | **29542947** | **1.14e-20** | **3** |
| **6** | **1** | **rs3104410** | **32683324** | **2.57e-20** | **2** |
| **6** | **1** | **rs1571878** | **167540842** | **3.21e-20** | **297** |
| **6** | **1** | **rs3104405** | **32682308** | **3.87e-20** | **0** |
| **6** | **1** | **rs114815710** | **30942121** | **4.19e-20** | **1** |
| **6** | **1** | **rs9272298** | **32603812** | **6.14e-20** | **16** |
| **6** | **1** | **rs79550239** | **32620628** | **1.1e-19** | **14** |
| **6** | **1** | **rs28366151** | **31592715** | **1.72e-19** | **11** |
| **6** | **1** | **rs9391724** | **31320795** | **1.72e-19** | **4** |
| **6** | **1** | **rs9268847** | **32429277** | **2.8e-19** | **0** |
| **6** | **1** | **rs76518703** | **31329466** | **3.62e-19** | **1** |
| **6** | **1** | **rs6932191** | **31385701** | **3.75e-19** | **19** |
| **6** | **1** | **rs9469053** | **31755776** | **3.77e-19** | **8** |
| **6** | **1** | **rs181174453** | **29767177** | **4.75e-19** | **133** |
| **6** | **1** | **rs62397979** | **31483206** | **4.92e-19** | **4** |
| **6** | **1** | **rs9501632** | **32420599** | **5.63e-19** | **5** |
| **6** | **1** | **rs1093** | **31321906** | **6.72e-19** | **9** |
| **6** | **1** | **rs115699278** | **31860832** | **1.4e-18** | **0** |
| **6** | **1** | **rs6905149** | **31944790** | **1.58e-18** | **3** |
| **6** | **1** | **rs3131004** | **31095294** | **4.18e-18** | **15** |
| **6** | **1** | **rs885950** | **31140152** | **4.19e-18** | **58** |
| **6** | **1** | **rs3915971** | **31269348** | **5.18e-18** | **6** |
| **6** | **1** | **rs72866766** | **31321184** | **5.43e-18** | **3** |
| **6** | **1** | **rs7769924** | **29443926** | **6.85e-18** | **33** |
| **6** | **1** | **rs1009382** | **32026107** | **7.32e-18** | **8** |
| **5** | **1** | **rs7731626** | **55444683** | **9.79e-18** | **4** |
| **6** | **1** | **rs17201826** | **32195021** | **1.86e-17** | **4** |
| **6** | **1** | **rs16895070** | **29521557** | **2.27e-17** | **31** |
| **6** | **1** | **rs733210** | **33082486** | **2.42e-17** | **54** |
| **6** | **1** | **rs9277531** | **33054687** | **3.23e-17** | **549** |
| **6** | **1** | **rs34277309** | **31329245** | **4.58e-17** | **5** |
| **6** | **1** | **rs2523995** | **30102184** | **5.62e-17** | **21** |
| **2** | **1** | **rs11889341** | **191943742** | **6.11e-17** | **16** |
| **6** | **1** | **rs111853141** | **31269251** | **9.43e-17** | **75** |
| **6** | **1** | **rs2507978** | **31351664** | **1.21e-16** | **1** |
| **6** | **1** | **rs28570682** | **30021740** | **1.61e-16** | **42** |
| **6** | **1** | **rs3130396** | **30223490** | **2.51e-16** | **3** |
| **6** | **1** | **rs4713235** | **29670478** | **2.65e-16** | **72** |
| **6** | **1** | **rs29243** | **29599102** | **3.21e-16** | **0** |
| **6** | **1** | **rs114880503** | **29856402** | **7.17e-16** | **0** |
| **6** | **1** | **rs9380374** | **33677556** | **7.61e-16** | **39** |
| **6** | **1** | **rs423639** | **32987774** | **8.87e-16** | **47** |
| **6** | **1** | **rs2853915** | **31275289** | **1.01e-15** | **1** |
| **6** | **1** | **rs9258191** | **29698036** | **1.61e-15** | **75** |
| **6** | **1** | **rs28744237** | **30991674** | **2.13e-15** | **6** |
| **6** | **1** | **rs35139945** | **32626267** | **2.72e-15** | **1** |
| **6** | **1** | **rs2523661** | **31439913** | **2.86e-15** | **6** |
| **6** | **1** | **rs9461247** | **26381917** | **3.72e-15** | **38** |
| **6** | **1** | **rs113355384** | **30874665** | **4.44e-15** | **4** |
| **6** | **1** | **rs9283890** | **31308412** | **4.86e-15** | **10** |
| **6** | **1** | **rs16870693** | **32711691** | **5.13e-15** | **0** |
| **6** | **1** | **rs115070292** | **29530974** | **5.46e-15** | **0** |
| **6** | **1** | **rs28749541** | **29947532** | **8.2e-15** | **9** |
| **6** | **1** | **rs2246871** | **31481146** | **8.73e-15** | **2** |
| **6** | **1** | **rs113243085** | **33516949** | **1.14e-14** | **6** |
| **6** | **1** | **rs2233955** | **31081251** | **1.18e-14** | **1** |
| **6** | **1** | **rs201082719** | **26321792** | **1.54e-14** | **0** |
| **6** | **1** | **rs3130663** | **30666594** | **1.76e-14** | **16** |
| **6** | **1** | **rs115493740** | **32838539** | **2.33e-14** | **2** |
| **6** | **1** | **rs3857549** | **26406053** | **2.34e-14** | **16** |
| **6** | **1** | **rs2859448** | **31436273** | **2.55e-14** | **1** |
| **6** | **1** | **rs10807036** | **28606397** | **4.2e-14** | **15** |
| **6** | **1** | **rs3893538** | **29924425** | **5.8e-14** | **2** |
| **6** | **1** | **rs499606** | **32194308** | **8.62e-14** | **11** |
| **6** | **1** | **rs7743500** | **26852053** | **8.77e-14** | **1** |
| **6** | **1** | **rs181997** | **32900718** | **9.71e-14** | **0** |
| **6** | **1** | **rs41287838** | **30931921** | **1.26e-13** | **24** |
| **6** | **1** | **rs614549** | **31840625** | **1.34e-13** | **3** |
| **6** | **1** | **rs35645205** | **33784797** | **1.36e-13** | **51** |
| **6** | **1** | **rs1383258** | **32783405** | **1.4e-13** | **3** |
| **6** | **1** | **rs74505854** | **27984726** | **1.83e-13** | **3** |
| **7** | **1** | **rs3757387** | **128576086** | **2.37e-13** | **8** |
| **6** | **1** | **rs858983** | **27173516** | **2.42e-13** | **0** |
| **6** | **1** | **rs28362859** | **44228815** | **3.92e-13** | **19** |
| **6** | **1** | **rs144112342** | **32858077** | **4.25e-13** | **0** |
| **6** | **1** | **rs3132597** | **30782780** | **4.34e-13** | **3** |
| **8** | **1** | **rs2618444** | **11338370** | **5.19e-13** | **15** |
| **6** | **1** | **rs2308931** | **33036950** | **5.28e-13** | **20** |
| **6** | **1** | **rs9276091** | **32696754** | **5.5e-13** | **54** |
| **6** | **1** | **rs16897791** | **30644359** | **6.63e-13** | **36** |
| **6** | **1** | **rs3094600** | **31347144** | **1.22e-12** | **1** |
| **6** | **1** | **rs9258224** | **29712256** | **1.55e-12** | **12** |
| **6** | **1** | **rs9267513** | **31569275** | **1.99e-12** | **0** |
| **6** | **1** | **rs68191** | **33480738** | **2.31e-12** | **13** |
| **6** | **1** | **rs9380069** | **28203300** | **3.36e-12** | **66** |
| **6** | **1** | **rs683208** | **32937901** | **4.4e-12** | **16** |
| **6** | **1** | **rs2596475** | **31326324** | **4.77e-12** | **6** |
| **6** | **1** | **rs3131063** | **30763756** | **4.81e-12** | **5** |
| **6** | **1** | **rs449635** | **33024166** | **2.83e-16** | **0** |
| **6** | **1** | **rs3869144** | **31831159** | **3.09e-16** | **2** |
| **6** | **1** | **rs10807081** | **31169764** | **4.5e-17** | **6** |
| **6** | **1** | **rs9266669** | **31348077** | **2.84e-13** | **111** |
| **6** | **1** | **rs35139284** | **32561370** | **1.37e-13** | **71** |
| **6** | **1** | **rs1130456** | **32627379** | **8.66e-14** | **71** |
| **6** | **1** | **rs7745002** | **32615421** | **6.7e-15** | **4** |
| **6** | **1** | **rs3104412** | **32585967** | **1.82e-15** | **204** |
| **9** | **1** | **rs1333048** | **22125347** | **5.67e-30** | **73** |

**Supplementary Table S6.Pleiotropic GenomicLoci in RA Identified by FUMA Using PLACO Results.**

| GenomicLocus | uniqID | rsID | chr | Lead SNP |  | N.SNPs | nIndSigSNPs | *p*_GWAS_ | **_Nearest gene_** |
| --- | --- | --- | --- | --- | --- | --- | --- | --- | --- |
| 1 | 1:2501222:G:T | rs10797431 | 1 | rs10797431 |  | 139 | 1 | 1.064e-08 | TNFRSF14/FAM213B/MMEL1/ TTC34 |
| 2 | 1:114377568:A:G | rs2476601 | 1 | rs2476601 |  | 298 | 7 | 6.251e-34 | PHTF1/RSBN1/PTPN22 |
| 3 | 2:191943742:C:T | rs11889341 | 2 | rs11889341 |  | 34 | 3 | 2.709e-17 | STAT4 |
| 4 | 5:55444683:A:G | rs7731626 | 5 | rs7731626 |  | 12 | 4 | 9.774e-18 | ANKRD55 |
| 5 | 6:29542947:C:T | rs3025650 | 6 | rs79774308  ;rs407934;  rs4481423;  rs60645096;  rs13202688;  rs73393463;  rs4476815;  rs201082719;  rs3757142;  rs3846841;  rs3025650;  rs1233380;  rs3131857  rs9257888; | rs858983;  rs9380069;  rs1778477;  rs449074;  rs11966698;  rs10484547;  rs1233478; | 1923 | 45 | 5.044e-21 | UBD/ GABBR1/ OR2H2 |
| 6 | 6:33677556:A:C | rs9380374 | 6 | rs115552552;rs538981518;rs68191;rs113243085; rs9368779;rs4713748 | rs210157;rs9380374;rs145044106;rs35645205; | 438 | 26 | 4.087e-16 | ITPR3/ UQCC2/ IP6K3 |
| 7 | 6:44228815:A:C | rs28362859 | 6 | rs28362859 |  | 30 | 2 | 9.897e-13 | HSP90AB1/SLC35B2/NFKBIE/TMEM/RP11444E17.6/TCTE1/AARS2 |
| 8 | 6:138228520:C:T | rs9494894 | 6 | rs9494894 |  | 44 | 1 | 2.484e-08 | TNFAIP3 |
| 9 | 6:167541258:C:G | rs3093017 | 6 | rs3777722; | rs3093017 | 472 | 13 | 4.336e-20 | FGFR1OP/ CCR6 |
| 10 | 7:50353192:A:G | rs10261758 | 7 | rs10261758 |  | 3 | 1 | 1.466e-08 | IKZF1 |
| 11 | 7:128576086:C:T | rs3757387 | 7 | rs3757387 |  | 14 | 1 | 1.466e-13 | IRF5 |
| 12 | 8:11337587:C:T | rs2409780 | 8 | rs2409780 |  | 26 | 1 | 5.374e-12 | FAM167A/ BLK |
| 13 | 10:31350494:A:G | rs1776616 | 10 | rs1776616 |  | 85 | 1 | 2.408e-09 | ZNF438 |
| 14 | 10:63779871:C:T | rs71508903 | 10 | rs71508903 |  | 11 | 1 | 2.536e-08 | ARID5B |
| 15 | 11:128488322:A:G | rs2156698 | 11 | rs2156698 |  | 9 | 1 | 1.437e-08 | NA |
| 16 | 18:77160235:A:C | rs56376587 | 18 | rs56376587 |  | 4 | 1 | 3.768e-09 | NFATC1 |
| 17 | 19:10463118:C:G | rs34536443 | 19 | rs34536443 |  | 4 | 1 | 3.034e-08 | ZGLP1/ CTD2369P2.10/FDX1L/CTD2369P2.12/RAVER1/TYK2/CDC37/PDE4A/ |
| 18 | 21:45703244:A:G | rs111469885 | 21 | rs111469885 |  | 9 | 1 | 2.65e-08 | AIRE |
| 19 | 22:21916166:C:T | rs5754100 | 22 | rs5754100 |  | 110 | 1 | 9.204e-09 | RIMBP3C/ UBE2L3/ YDJC/CCDC |
| 20 | 22:39749981:A:G | rs5757628 | 22 | rs5757628 |  | 59 | 1 | 7.58e-10 | AL031590.1/RPL3/ SYNGR1 |

**Supplementary Table S7. Pleiotropic GenomicLoci in stroke Identified by FUMA**

| GenomicLocus | uniqID | rsID | chr | Lead SNP | N.SNPs | nIndSigSNPs | *p*_GWAS_ | **_Nearest gene_** |
| --- | --- | --- | --- | --- | --- | --- | --- | --- |
| 1 | 4:155525276:A:G | rs2066865 | 4 | rs2066865 | 20 | 1 | 9.65539e-10 | FGB/FGA/ FGG/ LRAT |
| 2 | 5:142507651:A:G | rs3776299 | 5 | rs3776299 | 6 | 1 | 3.43534e-08 | ARHGAP26 |
| 3 | 6:1365244:A:G | rs79318212 | 6 | rs79318212 | 49 | 1 | 4.04688e-08 | FOXF2 |
| 4 | 6:160985526:A:G | rs118039278 | 6 | rs118039278 | 7 | 1 | 1.96164e-08 | LPA |
| 5 | 7:150690176:C:T | rs3918226 | 7 | rs3918226 | 1 | 1 | 1.80256e-08 | PLG |
| 6 | 8:10669464:A:G | rs75946928 | 8 | rs75946928 | 101 | 1 | 7.32437e-09 | SOX7/ |
| 7 | 9:22124140:A:T | rs7857118 | 9 | rs3731239;rs7857118 | 190 | 9 | 4.74351e-34 | NA |
| 8 | 12:111932800:C:T | rs7137828 | 12 | rs7137828 | 31 | 1 | 2.69284e-08 | SH2B3/ATXN2/BRAP/ACAD10/ RP11162P23.2/ALDH2/MAPKAPK5/NAA25/  TRAFD1/HECTD4/RPL6/ PTPN11 |
| 9 | 16:81144488:C:T | rs7194342 | 16 | rs7194342 | 10 | 1 | 3.31009e-08 | NA |
| 10 | 19:11159096:G:T | rs12052201 | 19 | rs12052201 | 39 | 1 | 2.01104e-08 | SMARCA4 |

**Supplementary Table S8.GWAS candidate genes of RA identified by FUSION**

| **Gene ID** | **Gene name** | **Z score** | ***p*** |
| --- | --- | --- | --- |
| ENSG00000157870 | FAM213B | 3.5496 | 3.86e-04 |
| ENSG00000116691 | MIIP | -3.5796 | 3.44e-04 |
| ENSG00000186715 | MST1L | -3.7209 | 1.99e-04 |
| ENSG00000117115 | PADI2 | 4.2972 | 1.73e-05 |
| ENSG00000204084 | INPP5B | 3.5831 | 3.40e-04 |
| ENSG00000273110 | RP1 | -3.6364 | 2.77e-04 |
| ENSG00000026297 | RNASET2 | -5.65929 | 1.52e-08 |
| ENSG00000197146 | AL133458 | -6.31834 | 2.64e-10 |
| ENSG00000128604 | IRF5 7 | 5.4473 | 5.11e-08 |
| ENSG00000183621 | ZNF438 | -5.3556 | 8.53e-08 |
| ENSG00000197728 | RPS26 | -3.6104 | 0.000306 |
| ENSG00000133103 | COG6 | 5.2424 | 1.58e-07 |
| ENSG00000198064 | RP11-347C12 | 3.9148 | 9.05e-05 |
| ENSG00000239697 | TNFSF12 | 3.61412 | 0.000301 |
| ENSG00000185651 | UBE2L3 | 4.4612 | 8.15e-06 |
| ENSG00000100201 | DDX17 | 4.2026 | 2.64e-05 |
| ENSG00000100321 | SYNGR1 | 4.7701 | 1.84e-06 |

**Supplementary Table S9.GWAS candidate genes of Stroke identified by FUSION**

| **Gene ID** |  | **Gene name** | **Z score** | ***p*** |
| --- | --- | --- | --- | --- |
| ENSG00000134222 |  | PSRC1 | -4.577221 | 4.71e-06 |
